# Association of intraoperative cerebral and somatic tissue oxygen saturation with postoperative acute kidney injury in adult patients undergoing multiple valve surgery

**DOI:** 10.1186/s12871-023-02279-7

**Published:** 2023-09-19

**Authors:** Hui Zhang, Taoyuan Zhang, Lihong Hou, Jing Zhao, Qianqian Fan, Lini Wang, Zhihong Lu, Hailong Dong, Chong Lei

**Affiliations:** Department of Anesthesiology and Perioperative Medicine, Xijing Hospital, Xi’an, 710032 China

**Keywords:** NIRS, Tissue oxygen saturation, Acute kidney injury, Multiple valve surgery

## Abstract

**Background:**

The association between tissue oxygenation with postoperative acute kidney injury (AKI) in adult patients undergoing multiple valve surgery has not been specifically studied.

**Methods:**

In this prospective exploratory cohort study, 99 patients were enrolled. The left forehead, the left forearm, the left upper thigh, and the left renal region tissue oxygen saturation using near-infrared spectroscopy were monitored. The association between each threshold and AKI was assessed. The relative and absolute thresholds were < 70%, < 75%, < 80%, < 85%, < 90%, < 95%, and < 100% baseline, and baseline-standard deviation (SD), -1.5 SD, -2 SD, -2.5 SD, and -3 SD. Multivariate logistic regression analysis was adopted to explore the association.

**Results:**

AKI occurred in 53 (54%) patients. The absolute value-based SrrO2 thresholds associated with AKI were baseline-3 SD (odds ratio [OR], 4.629; 95% confidence interval [CI], 1.238–17.314; *P* = 0.023) and baseline-2.5 SD (OR, 2.842; 95% CI, 1.025–7.881; *P* = 0.045) after adjusting for the potential confounders, those are renal region tissue oxygen saturation of 55% and 60%, but not statistically significant after correcting for multiple testing (corrected *P* = 0.114 and 0.179, respectively).

**Conclusion:**

The SrrO2 desaturation, defined as < baseline – 2.5 SD or < baseline – 3 SD, may be associated with AKI. The thresholds need to be verified in future large-scale studies.

**Trial registrations:**

The study was registered at ClinicalTrials.gov, first trial registration: 26/10/2017, identifier: NCT03323203.

## Introduction

Acute kidney injury (AKI) is a common and severe complication following cardiac surgery, especially in procedures involving prolonged cardiopulmonary bypass (CPB) [1]. AKI is known to be associated with a higher risk of postoperative complications and mortality [2, 3]. Current guidelines emphasize the importance of identifing high-risk patients and implementing early detection and prevention strategies to improve patient outcomes [4].

The diagnostic criteria for AKI, Kidney Disease: Improving Global Outcomes (KDIGO) [5], rely on changes in serum creatinine (S_Cr_) levels. Unfortunately, the delayed rise in S_Cr_ level after AKI develops, along with the complexity and intermittent nature in measuring renal injury biomarkers such as liver fatty acid binding protein (LFABP), cystatin C (CysC), neutrophil gelatinase-associated apolipoprotein (NGAL), etc. [6], makes them impractical for point-of-care monitoring and early detection of AKI.

The etiology of cardiac surgery-associated AKI (CSA-AKI) is often linked to decreased oxygen delivery and hemodynamic changes during CPB [7, 8]. Near-infrared spectroscopy (NIRS) is a technology used to assess tissue oxygenation by measuring regional oxyhemoglobin saturation (rSO_2_) in a non-invasive, continuous, and real-time manner [9, 10]. Consequently, continuous tissue oximetry is considered more clinically practical for perioperative monitoring and the early detection of CSA-AKI when compared to traditional biomarkers. While studies have reported an association between decreased renal oximetry and postoperative AKI in pediatric patients with congenital heart disease [11–13], the accuracy of NIRS in reflecting tissue oxygenation may be compromised in adult patients with increased tissue depth [14]. During CPB, oxygenation impairment is systemic rather than being limited to specific region [15]. Therefore, oximetry monitoring oxygenation in other more superficial tissue beds, such as the cerebral or muscle, may provide insights into kidney oxygenation.

In this prospective exploratory cohort study, the association of tissue oximetry in different tissue beds (e.g., the forehead, the left arm, the left leg, and the left kidney) with postoperative AKI was explored in patients undergoing multiple valve surgery (CPB ≥ 90 min). It was assumed that changes in oxygenation of cerebral, peripheral, or renal tissue bed might be associated with the incidence of CSA-AKI. If the association was confirmed, the tissue oximetry could be used as a point-of-care and continuous monitoring technique for perioperative AKI detection.

## Methods

The Institutional Review Board of Xijing Hospital (Xian, China) approved the study, and the study was registered at ClinicalTrials.gov (first trial registration: 26/10/2017, identifier: NCT03323203). The research protocol was consistent with the principles outlined in the Declaration of Helsinki. The trial was conducted at Xijing Hospital (a tertiary teaching hospital). Both verbal and written informed consent were obtained from patients or their legal representatives before surgery.

### Patients

Between January 2018 and November 2019, patients aging above 18 years old who were scheduled for elective multiple valve surgery were screened for eligibility. None of patients needed oxygen supplementation before surgery. Patients with 1) trauma, deformity, or other abnormalities on the sensor placement site, which might affect the accuracy of monitoring; 2) preoperative renal failure requiring renal replacement therapy; 3) preoperative intubation requiring mechanical ventilation; 4) mental disorders and being unable to cooperate; 5) preoperative blood transfusion; and 6) patients scheduled for emergency or urgent surgery were excluded from this study.

### Anesthesia and perioperative care

After entering the operating room, patients were first connected to standard monitoring, including electrocardiography (ECG), pulse oximetry, and left radical arterial blood pressure. Anesthesia was induced with a bolus injection of 0.02–0.04 mg/kg midazolam, 0.1–0.2 mg/kg etomidate, 0.5–1 µg/kg sufentanil, followed by 0.6–0.9 mg/kg rocuronium after the loss of consciousness. Then, intubation and mechanical ventilation were carried out. The mechanical ventilation mode was volume-controlled ventilation with a tidal volume of 6–8 ml/kg, a respiratory rate of 12–14 breaths/min, and a fraction of inspired oxygen (FiO_2_) of 50%. After that, a central line was accessed via the right jugular vein. During the surgery, the bispectral index was maintained at 40 to 60. The anesthesia was maintained with continuous infusion of propofol or inhaled sevoflurane. After administration of heparin (400 IU/kg) for 5 min, CPB was started after waiting for the activated clotting time to 480 s. During CPB, the extracorporeal circulation speed was maintained at 2.0–2.8 L/kg/m^2^, so that the mean arterial pressure (MAP) was in the range of 50–80 mmHg. After CPB, heparin was antagonized with protamine sulfate. After the procedure, all patients were transferred to the cardiac intensive care unit (CICU) and care was provided by physicians and nurses in the CICU who were not involved in the present study.

### Tissue oxygenation monitoring

Tissue oxygenation was monitored using a near-infrared spectroscopy-based tissue oximeter (FORE-SIGHT Elite, CAS Medical System Inc., New York, NY, USA). In the present study, tissue oxygenation of four different tissue beds was monitored, including S_ct_O_2_ on the left forehead, the left forearm over the brachioradialis muscle (S_arm_O_2_), left upper thigh over the quadriceps (S_leg_O_2_), and the left renal region (S_rr_O_2_). NIRS probe was applied to the left side of the renal area to monitor S_rr_O_2_ under sonographic guidance (Mindray, M-7, probe C 5–1 s, frequency 1–5 MHz) to identify the renal region closest to the skin. The depth of the kidney (distance from the skin surface to the kidney capsule) was measured on the long-axis image of the kidney. All four probes were placed before tracheal intubation when the patient was breathing at FiO_2_ of 0.21, and the acquired data were recorded as a baseline value. The monitoring and data recording were started and were stopped at the end of surgery. The tissue oximetry recorded the oximetry data every two seconds.

### Tissue desaturation (exposure)

The main objective of the present research was to explore the association between tissue desaturation measured at different tissue beds (i.e., left S_ct_O_2_, S_arm_O_2_, S_leg_O_2_, and S_rr_O_2_) and the incidence of AKI. The degree of tissue desaturation may depend on magnitude and duration rather than changes in individual time points [16, 17]. Therefore, the association between each AUT (area under the threshold) and the incidence of AKI was investigated. AUT (min × %) was the product of the difference between the actual measurement value and the threshold value and the time when the actual measurement value exceeded the threshold. The thresholds used were < 70%, < 75%, < 80%, < 85%, < 90%, < 95%, and < 100% by referring to the baseline. This was the relative change. In addition, the absolute changes were assessed, and the thresholds were defined as baseline-SD, baseline-1.5 SD, baseline-2 SD, baseline-2.5 SD, and baseline-3 SD.

### Outcomes

The primary outcome measure was the incidence of AKI according to the KDIGO criteria [5], which defined as an increase in S_Cr_ 0.3 mg/dl (26.5 mol/L) within 48 h, or an increment in S_Cr_ to 1.5 times of baseline within 7 days, or urinary volume < 0.5 ml/kg/h for 6 h.

Other outcomes included the length of mechanical ventilation, the length of CCU stay, the length of hospital stay, and adverse events at the time of hospital discharge and at 30 days after surgery. The relationships between tissue oxygenation and non-renal outcomes in different tissue beds were also explored.

### Clinical covariates

Patients’ demographic data were collected, including age, gender, body mass index (BMI), and American Society of Anesthesiologists (ASA) physical status score. Medical history included stroke, hypertension, diabetes mellitus, dyslipidemia, peptic ulcer disease, gastrointestinal bleeding, peripheral vascular disease, coronary artery disease, coagulopathy, chronic obstructive pulmonary disease (COPD), and chronic kidney disease. Surgical data included operation time, CPB time, volume of crystalloid and colloid transfusion, intraoperative minimum hemoglobin level, urinary volume, blood loss, and blood transfusion. The intraoperative hemodynamics and arterial blood gas were also recorded.

### Statistical analysis

This study was conducted as an investigator-initiated pilot study to explore the.

feasibility of the coherence effect. Due to its exploratory nature (heterogeneous and sparse data on the efficacy of different tissue oxygen saturation in multiple valve replacement patients to predict AKI) and inestimable recruitment variables (patient flow and preoperative condition), no specific assumptions could be made regarding the effect size of the intervention. The analyses are, therefore, explorative; p values should be interpreted as such.

Continuous variables were expressed as mean (SD) or median (interquartile range (IQR)). The difference between patients with AKI and those without AKI was tested using the parametric unpaired Student’s t-test or the non-parametric Mann–Whitney U test (as appropriate). Categorical variables were described as frequency (%).

The associations between tissue desaturation and the incidence of AKI were analyzed using multivariate logistic regression analysis adjusted for confounders. For multivariate logistic regression analysis, variables were selected on the basis of previous findings and clinical constraints, i.e., Euroscore II, intraoperative inotropic score, length of CPB, the lowest MAP during CPB, and torsemide use during CPB [18, 19]. The baseline characteristics show that over 66% of patients with NYHA grade III, which may be a confounder in this study but also have collinearity with Euroscore II, so we don’t include it in logistic regression analysis. For tissue oxygenation at different tissue beds, two different types of thresholds (e.g., absolute changes and relative values) were explored. Up to seven different tests were performed for each type of threshold. In order to keep the type I error at 5%, P value correction was performed for each set of tests for each tissue bed and each threshold type using the Holm-Bonferroni method. The statistical analysis was carried out using SAS 9.1.4 software (SAS Institute Inc., USA). The significance level of each general assumption was set to 0.05.

## Results

### Patients’ characteristics and perioperative data

**Fig. 1:**
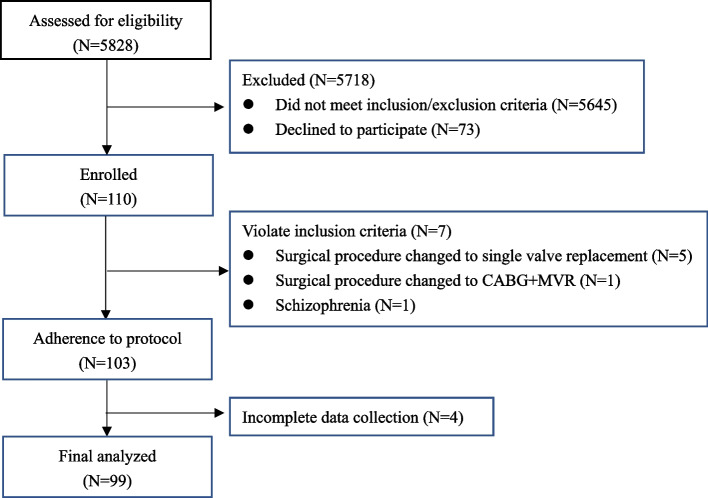
Study flow chart. MVR, mitral valve replacement; CABG, coronary artery bypass grafting

Of the 5828 patients screened, 110 were enrolled. After excluding cases with missing tissue oximetry data, 99 cases were finally analyzed (Fig. 1). Patients aged 54.0 ± 7.0 years old, and 57 (57.6%) patients were male. Patients’ characteristics, comorbidities, and regular medications are summarized in Table 1.

**Table 1:** Patient characteristics, clinical information and tissue oxygenation of the study population (*n* = 99)

Variables	Patients (*n* = 99)
***Patient characteristics***	
Age (yr)	54.0 ± 7.6
Sex = male (*n* (%))	57 (57.6)
BMI (kg/m2)	22.9 ± 3.4
NYHA = III (*n* (%))	66 (66.7)
Preoperative Cr (umol/L)	86.1 ± 24.3
LVEF (%)	52.6 ± 5.9
Euroscore II (%)	1.18 (0.90, 1.75)
Depth of kidney (cm)	3.3 ± 0.9
***Comorbidities***	
Atrial fibrillation (*n* (%))	55 (55.6)
Pulmonary Hypertension (*n* (%))	35 (35.4)
Coronary artery disease (*n* (%))	9 (9.1)
Congestive heart failure (*n* (%))	5 (5.1)
Stroke (*n* (%))	6 (6.1)
Diabetes (*n* (%))	5 (5.1)
Hypertension (*n* (%))	10 (10.1)
Peptic ulcer disease (*n* (%))	3 (3.0)
Dyslipidemia (*n* (%))	7 (7.1)
COPD (*n* (%))	2 (2.0)
Smoking (*n* (%))	32 (32.7)
PCI history (*n* (%))	4 (4.1)
PBMV history (*n* (%))	0 (0)
***Current Regular Medication***	
Beta-blocker (*n* (%))	17 (17.2)
Calcium channel blocker (*n* (%))	11 (11.1)
Diuretic (*n* (%))	91 (91.9)
Anti-arrhythmic (*n* (%)	4 (4.0)
Digoxin (*n* (%)	34 (34.3)
Nitroglycerine (*n* (%)	7 (7.1)
Statin (*n* (%))	15 (15.2)
Inotropic drugs (*n* (%))	17 (17.2)
Vasodilator (*n* (%))	15 (15.2)
Proton pump inhibitor (*n* (%))	10 (10.1)
Low molecular weight heparin (*n* (%))	4 (4.0)

*BMI* body mass index, *NYHA* New York Heart Association, *LVEF* left ventricular ejection fraction, *COPD* chronic obstructive pulmonary disease, *PCI* percutaneous transluminal coronary intervention, *PBMV* percutaneous balloon mitral valvotomy

Data are in mean (SD) or median (interquartile range) for continuous variables and in count (*n*) and percentage (%) for binary variables

### Univariate analysis of clinical covariates with postoperative AKI

A total of 53 patients, accounting for 54% of the study population, developed AKI following cardiac surgery. This finding aligns with our previous report (64% [20]), a recent report focused on the Korean population (40.1% [21]), as well as studies conducted on the MIMIC-III cohort (58% with severe stage 2 or 3 AKI [22]) and the eICU cohort (37% with severe stage 2 or 3 AKI [22]). In patients with AKI, the operation time was 281.6 ± 74.4 min, which was significantly longer than that in individuals without AKI (241.9 ± 44.9 min, *P* = 0.002). Patients with AKI tended to receive more Torsemide 10 (10, 10) mg than those without AKI (10 (0, 10) mg) during CPB (P = 0.016), more inotropic drugs (5.0 (5.0, 8.0) vs. 5.0 (4.0, 5.0), *P* = 0.002), and more crystalloid (700 (700, 800) vs. 700 (700,700) ml, *P* = 0.009) during surgery. After surgery, patients who developed postoperative AKI needed longer mechanical ventilation (26.5 (21.0, 45.1) vs. 22.3 (17.8, 25.0) h, *P* = 0.0006), more packed red blood cell transfusion (130 (0, 290) vs. 0 (0, 0) ml, *P* = 0.002), and crystalloid infusion (2639.0 (2324.0, 3121.0) vs. 2342.5 (2043.0, 2659.0) ml, *P* = 0.021), and they had a longer CICU stay (68.0 (62.0, 90.0) vs. 46.1 (43.5, 68.5) h, *P* = 0.006). Patients with AKI had a higher incidence of postoperative complications, including significantly higher incidence rates of acute hepatic injury (34.0% vs. 6.5%, *P* = 0.001), pulmonary infection (62.3% vs. 41.3%, *P* = 0.037), and atelectasis (37.7% vs. 13.0%, *P* = 0.005) (Table 2). Besides, 4 patients in the study cohort died, and they experienced postoperative AKI.

**Table 2:** Univariate analysis of clinical covariates with postoperative acute kidney injury (AKI)

Variables	Non-AKI (*n* = 46)	AKI (*n* = 53)	*P-value*
***Surgical information***			
Surgical procedure (*n* (%))			0.747
AVR + MVR + TVR (*n* (%))	0 (0.0)	1 (1.9)	
AVR + MVR + TVP (*n* (%))	45 (97.8)	49 (92.5)	
AVP + MVP + TVP (*n* (%))	0 (0.0)	2 (3.8)	
AVR + MVR (n (%))	1(2.2)	1 (1.9)	
Cardiac ablation (*n* (%))	13 (28.3)	12 (22.6)	0.421
Length of surgery (min)	241.9 ± 44.9	281.6 ± 74.4	0.002
Intraoperative inotropic score	5.0 (4.0, 5.0)	5.0 (5.0, 8.0)	0.002
***Intraoperative Fluid management***			
Crystalloid (ml)	700 (700, 700)	700 (700, 800)	0.009
Colloid (ml)	0 (0, 0)	0 (0, 0)	0.367
PRBC (ml)	0 (0, 0)	0 (0, 0)	0.316
Platelets (ml)	0 (0, 0)	0 (0, 0)	0.352
FFP (ml)	0 (0, 340)	195 (0, 393)	0.279
Cryoprecipitate (ml)	0 (0, 0)	0 (0, 0)	0.185
Homologous RBC (ml)	500 (450, 700)	500 (500, 775)	0.379
Urine output (ml)	300 (200,550)	375 (200, 500)	0.638
Blood loss (ml)	400 (300, 450)	400 (300, 500)	0.150
***CPB course***			
Total CPB time (min)	141 (120, 162)	153 (120, 191)	0.166
Cross-clamp time (min)	92.5 (74.0, 115.0)	95.0 (71.0, 109.0)	0.891
Lowest Hb during CPB (g/dL)	7.7 (7.0, 9.1)	8.4 (7.4, 9.1)	0.122
Lowest MAP during CPB (mmHg)	41.5 (34.0, 46.0)	40.0 (36.0, 46.0)	0.872
Lowest temperature during CPB (℃)	31.0 ± 0.7	30.9 ± 0.9	0.612
Dopamine (mg)	0 (0, 0)	0 (0, 0)	0.271
Nonepinephrine (ug)	0 (0, 20)	0 (0, 25)	0.678
Epinephrine (ug)	0 (0, 0)	0 (0, 0)	0.807
Isoproterenol (ug)	0 (0, 0)	0 (0, 0)	0.170
Isosorbide (mg)	0 (0, 0)	0 (0, 0)	0.522
Aramine (mg)	0 (0, 2)	0 (0, 2)	0.515
Torasemide (mg)	10 (0, 10)	10 (10, 10)	0.016
Hydroprednisolone (mg)	0 (0, 100)	100 (0, 180)	0.210
PRBC (ml)	0 (0, 260)	0 (0, 240)	0.940
PRBC Storage duration (d)	11 (9, 11)	12 (9, 15)	0.318
FFP (ml)	0 (0, 0)	0 (0, 0)	0.418
Crystalloid (ml)	1000 (1000, 1000)	1000 (1000, 1000)	0.693
Colloid (ml)	1000 (1000, 1000)	1000 (1000, 1000)	0.423
Urine output (ml)	800 (350, 1200)	600 (300, 1000)	0.156
***CCU***			
APACHE II score	18 (18, 18)	18(18 18)	0.837
Intubation time (h)	22.3 (17.8, 25.0)	26.5 (21.0, 45.1)	0.0006
Length of CCU stay (hours)	46.1 (43.5, 68.5)	68.0 (62.0, 90.0)	0.006
Readmitted to CCU (n (%))	0 ( 0.0)	4 (7.6)	0.121
Furosemide (mg)	20 (0, 40)	20 (20, 40)	0.056
Hydrprednisone (mg)	0 (0, 0)	0 (0, 0)	0.627
Inotropic Score	8 (5, 10)	6.5 (3, 8)	0.250
Crystalloid (ml)	2342.5 (2043.0, 2659.0)	2639.0 (2324.0, 3121.0)	0.021
Colloid (ml)	0 (0, 0)	0 (0, 0)	0.222
Albumin (ml)	0 (0, 0)	0 (0, 0)	0.333
PRBC (ml)	0 (0, 0)	130 (0, 290)	0.002
PRBC Storage (d)	13 (8, 14)	12 (8, 15)	1.000
FFP (ml)	400 (352.5, 772.5)	700 (440, 960)	0.058
Urine output (ml)	3370 (2730, 3880)	3070 (2620, 3950)	0.352
Chest tube drainage (ml)	445 (325, 600)	350 (225,775)	0.431
***Length of hospital stay (d)***	11 ± 5	11 ± 4	0.811
***Postoperative complications***			
Death (*n* (%))	0 (0.0)	4 (7.5)	0.164
Reoperation (*n* (%))	2 (4.4)	2 (3.8)	1.000
New AF or flutter (*n* (%))	0 (0.0)	2 (3.8)	0.284
Stroke (*n* (%))	0 (0.0)	0 (0.0)	1.000
Acute hepatic injury (*n* (%))	3 (6.5)	18 (34.0)	0.001
**Pulmonary events** (*n* (%))			
Pulmonary infection (*n* (%))	19 (41.3)	33 (62.3)	0.037
Atelectasis (*n* (%))	6 (13.0)	20 (37.7)	0.005
Pleural effusion (*n* (%))	3 (6.5)	10 (18.9)	0.070
Pneumothorax (*n* (%))?	1 (2.2)	1 (1.9)	1.000
Cardiac dysfunction (*n* (%))	5 (10.9)	8 (15.1)	0.535
Gastrointestinal bleeding (*n* (%))	2 (4.3)	3 (5.7)	1.000
Respiratory failure (*n* (%))	5 (10.9)	13 (23.5)	0.079
Thrombosis (*n* (%))	0 (0.0)	2 (3.8)	0.284
Wound infection (*n* (%))	2 (4.4)	3 (5.7)	1.000
MODS (*n* (%))	0 (0.0)	5 (9.4)	0.093
Sepsis (*n* (%))	0 (0.0)	1 (1.9)	1.000
Postoperative day 3 delirium	4 (8.7)	3 (5.7)	0.846

*AVR* aortic valve replacement, *MVR* mitral valve replacement, *AVP* aortic valvoplasty, *MVP* mitral valvoplasty, *TVP* tricuspid valvoplasty, *PRBC* packed red blood cell, *FFP* fresh frozen plasma, *CPB* cardiopulmonary bypass, *Hb* hemoglobin, *MAP* Mean arterial pressure, *CCU* cardiac care unit, *APACHE* Acute Physiology and Chronic Health Evaluation, *AF*atrial fibrillation

Data are in mean (SD) or median (interquartile range) for continuous variables and in count (n) and percentage (%) for binary variables

### Multivariate logistic regression analysis of AUT and AKI

The confounding factors included in the multivariate logistic regression analysis were Euroscore II, intraoperative inotropic score, length of CPB, the lowest MAP during CPB, and Torsemide during CPB, which were selected on the basis of previous findings and clinical constraints [18, 19]. The absolute value-based SrrO2 thresholds associated with AKI were baseline -3 SD (odds ratio (OR), 4.629; 95% CI, 1.238, 17.314; *P* = 0.023) and baseline -2.5 SD (OR, 2.842; 95% CI, 1.025, 7.881; *P* = 0.045) after adjusting for the above-mentioned confounders (Table 3). The absolute value-based S_rr_O2 thresholds, baseline -3 SD and baseline -2.5 SD, were renal rSO2 values of 55% and 60%. These represent the approximate mean value of the baseline (77.8 ± 7.6%) minus 3 and 2.5 standard deviations (SDs), respectively.

**Table 3:** Multivariable analysis of AUT of absolute S_rr_O_2_ (baseline-3 SD) and AKI

Variables	OR (95% CI)	*P-value*
S_rr_O_2_ AUT (per min%, baseline -3SD)^a^	4.629 (1.238, 17.314)	0.023
Euroscore II (< 1.18 versus ≥ 1.18)	0.702 (0.276, 1.788)	0.458
Intraoperative inotropic score (< 5 versus ≥ 5)	2.228 (0.504, 9.860)	0.291
Length of CPB (< 140 min versus ≥ 140 min)	0.423 (0.168, 1.066)	0.068
Lowest MAP during CPB (per 5 mmHg increase)	0.935 (0.625, 1.398)	0.742
Torsemide during CPB (yes versus no)	0.189 (0.021, 1.724)	0.140

*Abbreviations*: *CI* confidence interval, *OR* odds ratio, *S*_*rr*_*O*_*2*_ renal region tissue oxygen saturation

The confounders included in this multivariable logistic regression are Euroscore II, intraoperative inotropic score, length of CPB, lowest MAP during CPB, and Torsemide during CPB

^a^When using the absolute S_rr_O_2_ desaturation (baseline-2.5 SD) in multivariable logistic regression, the OR is 2.84 (95% CI, 1.03, 7.88; *P* = 0.045)

### Association between the AUT and AKI

The incidence rates of AKI in patients who exceeded and did not exceed specific thresholds are presented in Table 4 for thresholds based on relative changes and Table 5 for thresholds based on absolute values. Renal region desaturation, which was defined by AUT < baseline -2.5 SD (OR, 2.842; 95% CI, 1.025, 7.881; *P* = 0.045, corrected *P* = 0.179) and by AUT < baseline – 3 SD (OR, 4.629; 95% CI, 1.238, 17.314; *P* = 0.023; correct *P* = 0.114), was correlated with S_rr_O_2_ desaturation in patients with AKI, while this correlation was not statistically significant after correcting for multiple testing (Table 5).

**Table 4:**
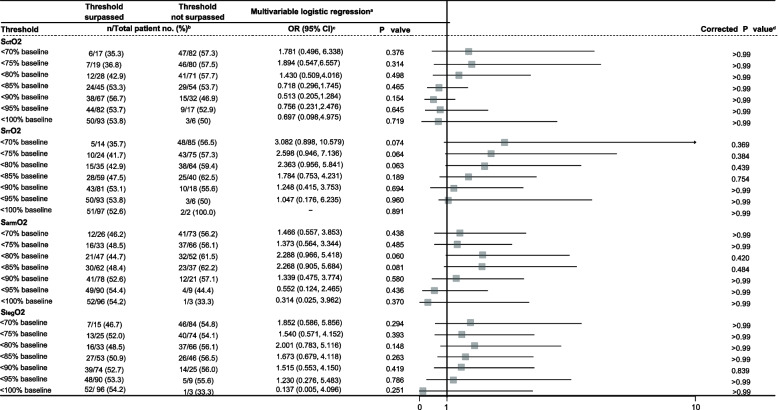
Incidence of relative change-based tissue desaturation and their associations with AKI

*Abbreviations*: - not assessable, *CI* confidence interval, *OR* odds ratio, *S*_*ct*_*O*_*2*_ cerebral tissue oxygen saturation, *S*_*rr*_*O*_*2*_, renal region tissue oxygen saturation, *S*_*arm*_*O*_*2*_ forearm tissue oxygen saturation, *S*_*leg*_*O*_*2*_ upper thigh tissue oxygen saturation

^a^The confounding factors included in the multivariable analysis are Euroscore II, intraoperative inotropic score, length of CPB, lowest MAP during CPB, and Torsemide during CPB

^b^The denominator is the number of patients who surpassed or did not surpass the threshold, while the numerator is the number of patients with AKI. Due to missing data

^c^The OR is the odds of AKI when desaturation occurs over the odds of AKI when desaturation does not occur. OR > 1 suggests desaturation is associated with an increased risk of AKI, whereas OR < 1 suggests desaturation is associated with a decreased risk of AKI

^d^The *P* values were corrected for multiple testing using the Holm-Bonferroni method for each tissue bed

**Table 5:**
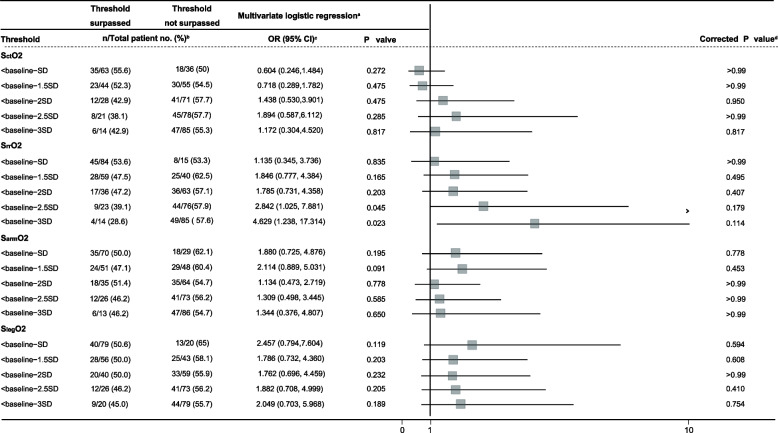
Incidence of absolute value-based tissue desaturation and their associations with AKI

*Abbreviations*: *CI* confidence interval, *OR* odds ratio, *SctO2* cerebral tissue oxygen saturation, *SrrO2* renal region tissue oxygen saturation, *SarmO2* forearm tissue oxygen saturation, *SlegO2* upper thigh tissue oxygen saturation

^a^The confounding factors included in the multivariable analysis are Euroscore II, intraoperative inotropic score, length of CPB, lowest MAP during CPB, and Torsemide during CPB

^b^The denominator is the number of patients who surpassed or did not surpass the threshold, while the numerator is the number of patients with AKI. Due to missing data

^c^The OR is the odds of AKI when desaturation occurs over the odds of AKI when desaturation does not occur. OR > 1 suggests desaturation is associated with an increased risk of AKI, whereas OR < 1 suggests desaturation is associated with a decreased risk of AKI

^d^The *P* values were corrected for multiple testing using the Holm-Bonferroni method for each tissue bed

None of the AUTs calculated by relative changes in threshold or absolute values were associated with the incidence of AKI according to S_ct_O_2_, S_rr_O_2_, S_arm_O_2,_ or S_leg_O_2_ data (Tables 4 and 5).

## Discussion

AKI occurred in 54% of patients undergoing multiple valve surgery. Such group of patients have higher risk of AKI and develop a worse prognosis due to multifactorial elements. Renal ischemia, reperfusion, inflammation, hemolysis, oxidative stress, cholesterol emboli, and toxins contribute to the development and progression of AKI [20, 23]. The SrrO2 desaturation, defined as AUT either < baseline – 2.5 SD or < baseline – 3 SD, that is renal rSO2 value of 55% or 60%, was correlated with the increased risk of AKI after adjusting for confounding factors. However, the above-mentioned data were not statistically significant after correcting for multiple testing. According to the threshold calculated by relative changes or absolute values, no AUT was associated with the incidence of AKI. The results of the present study suggested the potential of using < baseline- 2.5 SD of S_rr_O_2_ as the threshold of renal desaturation in cardiac patients undergoing multiple valve surgery. Although the threshold < baseline – 3 SD was correlated with the incidence of AKI in the present study, < baseline- 2.5 SD was adopted as the threshold for renal desaturation definition to avoid underestimation in the diagnosis of renal desaturation.

AKI is a frequent observed complication in patients with heart disease [24, 25]. Clinical practice has demonstrated a delayed response to intraoperative kidney injury. Patients undergoing cardiac surgery often encountered ischemia–reperfusion (IR) injury, which is considered to be a key factor to AKI [23]. Using renal micropuncture in rodent IR models revealed persistent preglomerular vasoconstriction and decreased regional blood flow to the outer medulla [26]. Tubular injury caused by ischemia and reduced oxygen delivery, can compromise the glomerular filtration rate by activating tubuloglomerular feedback. In rat models, 30-min ischemia resulted in reduced renal blood flow (RBF) and oxygenation that lasted for 3 h [27]. In swine models, 45 min of aortic cross-clamping led to a 4-h period of hypoxia [28]. Animal models using MRI have shown a global reduction in tissue oxygenation during episodes of ischemia [29]. As a result, if systemic physiological derangements occur, the oxygenation of tissue beds will be at risk. Near-infrared spectroscopy (NIRS) is a technology used to assess tissue oxygenation by measuring regional oxyhemoglobin saturation (rSO_2_) in a non-invasive, continuous, and real-time manner [9, 10]. In clinical settings, NIRS has been able to provide clinicians with potentially valuable information in patients with impaired microcirculation (systemic and cerebral). NIRS has progressed beyond the assessment of brain oxygenation to monitor local tissue and muscle oxygenation and perfusion in cardiac, vascular, and thoracic surgery [9]. Nowadays, tissue oximetry has been used more and more in other ways like renal region perfusion. An important relevant question that should be answered is, between different organs and tissue beds (e.g., tissue beds in the arm vs. leg vs. renal region), the change in oxygenation of which tissue bed(s) is correlated with renal tissue bed, and it can thus be used as a substitute for monitoring oxygenation of kidney tissue. Consequentially, the predictive power of cerebral oxygenation, arm oxygenation, leg oxygenation, and renal region oxygenation was evaluated. In this small cohort, no association between postoperative AKI and deoxygenation was found among all four absolute or relative thresholds of tissue deoxygenation.

Few studies have demonstrated that in infants who underwent cardiac surgery, prolonged low renal oxygen saturation values during surgery were associated with the development of postoperative AKI and might be superior to conventional biochemical markers [11, 13, 30]. Current evidence for predicting AKI in adult cardiac patients with tissue oxygenation is still inconsistent. A recent retrospective study investigated the relationship between renal region, cerebral, peripheral desaturation, and AKI in 59 patients (41 patients were finally analyzed due to the missing data of tissue oxygenation) who underwent coronary artery bypass grafting (CABG) with or without CPB [31]. It was attempted to investigate the ability of intra-operative renal region tissue oxygenation (S_rt_O2) to predict postoperative AKI and compare its predictive power with that of peripheral vascular and cerebral tissue oxygen saturation (S_pt_O2 and S_ct_O2, respectively). In this small retrospective study, no association was identified between tissue oxygenation and cerebral oxygenation measured in the renal area and postoperative AKI, which was consistent with the findings of the present study. In contrast, a S_pt_O2 decrease of > 10% from baseline was a reasonable predictor, and it was explained that S_pt_O2 could be a better indicator of systemic tissue oxygenation, which was not shown in the present study. However, renal depth was not recorded in this study, which might explain the lack of predictive value in AKI. In contrast, the renal depth was recorded in the present study (3.3 ± 0.9 cm), which was theoretically reachable for oxygenation monitoring. In a prospective observational study, 95 adult patients undergoing elective valvular surgery had an absolute decrease of less than 55% in renal zone oxygenation S_rt_O2, which was significantly associated with the risk of postoperative kidney damage [32]. Of note, the average depth of the right kidney in the study population was 29.7 mm and that of the left kidney was 30.6 mm, which are less than the renal depth achieved in the present study (33 mm). Another prospective observational study attempted to evaluate whether brain and muscle (thenar muscle) oxygenation measured by NIRS could predict the risk of AKI in addition to cystatin C and NGAL concentrations in 114 patients undergoing cardiac surgery using CPB [33]. They found that the most accurate predictor of AKI was the NIRS recorded at 20 min after CPB (cutoff ≤ 54.5% for muscle and ≤ 62.5% for brain). However, only absolute NIRS value was used, and NIRS data were reported at discrete time points, rather than at the AUT of NIRS. Comparably, patients underwent multiple valve surgery in the present study, multiple thresholds were explored, including relative and absolute data, and, importantly, the accumulative effect of tissue desaturation was investigated in lieu of at a single time point. Another study comparing local oxygen saturation in the thighs, forehead, and abdomen also found that tissue oxygen saturation in the thighs was an independent risk factor for AKI [34, 35], no matter in pediatric or adult patients undergoing cardiac surgery, suggesting that body oxygen saturation may be a better indicator of "general" tissue oxygen saturation status because muscle vasculature is more sensitive to vasoconstrictors compared with brain. However, further in-depth research is required to confirm and explore this finding.

It is noteworthy that NIRS measures oxygen saturation levels in local tissues up to 3 to 4 cm below commercially available sensors [14]. At present, NIRS may not measure regional tissue oxygen saturation in the true renal region (renal cortex and medulla). NIRS can measure rSO_2_ in the renal cortex in patients with renal depth < 30 mm. However, the measurement of renal depth > 30 mm rSO_2_ may be interfered by the perirenal adipose tissue surrounding the kidney. In addition, AKI may occur postoperatively due to several clinical factors, which were not assessed in the present study. This may partially explain deficiencies in the results of the present study.

In the present study, the oxygenation reference value was defined as the valve before tracheal intubation on the room air. Baseline values after induction of general anesthesia may be more representative of physiological conditions during surgery than awake baseline values and are therefore more likely to be the basis for determining the occurrence of associated adverse events [36]. In the aforementioned study [31], baseline values were defined as the average of data recorded within 5 min before commencing surgery, and it was attempted to explore the most significant decrease in S_rr_O_2_ during surgery and the degree of S_rr_O_2_ was associated with operation time. The present study and another study [32] used pre-intubation as the baseline and showed no or minor differences in oxygenation changes.

The present study has several limitations that should be acknowledged. Firstly, the small sample size may have limited the statistical power and precision of the estimated odds ratios, as evidenced by the wide range of confidence intervals. Moreover, due to the observational nature of the study and the small sample size, potential confounding factors may not have been adequately adjusted for. Secondly, the current study did not explore the dose-dependent association between tissue oximetry and the severity of AKI, primarily due to the limited sample size and limited number of events observed. Thirdly, the study was conducted in a single center and limited enrollment to valvular procedures, which enhanced internal validity but may compromise the external validity and generalizability of the findings. Fourthly, since there are various tissue oximeters approved by the Food and Drug Administration, it is uncertain whether the cutoff value determined in this study can be universally applied when using other products. In summary, the current study should be interpreted as a proof-of-concept and exploratory study. The findings warrant further validation in larger multi-center trials across various clinical scenarios before considering their application in clinical practice. Furthermore, future studies should prioritize determining the most sensitive sites for tissue oxygenation monitoring and identifying optimal cutoff values for early detection of AKI. It would be crucial to investigate whether early initiation of preventive and treatment strategies based on continuous point-of-care oximetry monitoring can provide clinical benefits for high-risk patients and improve their prognosis. Understanding the potential impact of early interventions on patient outcomes is essential for guiding clinical practice and optimizing AKI management in at-risk populations.

## Conclusions

In conclusion, AKI occurred in 54% of patients undergoing multiple valve surgery. The SrrO2 desaturation, defined as AUT either < baseline – 2.5 SD or < baseline – 3 SD, could increase the risk of AKI. Further research is required to clarify the generalizability of the findings for other populations and the use of SrrO2 desaturation as a potential indicator for AKI.

## Data Availability

The datasets used and/or analyzed during the current study are available from the corresponding author on reasonable request.
